# Outcome Predictors of Oral Food Challenge in Children

**DOI:** 10.3390/children13010146

**Published:** 2026-01-20

**Authors:** Vojko Berce, Anja Pintarič Lonzarić, Elena Pelivanova, Sara Jagodic

**Affiliations:** 1 Department of Pediatrics, University Medical Centre Maribor, Ljubljanska Ulica 5, 2000 Maribor, Slovenia; anjapintariclonzaric@gmail.com; 2 Department of Internal Medicine, University Medical Centre Maribor, Ljubljanska Ulica 5, 2000 Maribor, Slovenia; elena.pelivanova@yahoo.com; 3 Department of Pediatrics, General Hospital Ptuj, Potrčeva Cesta 23, 2250 Ptuj, Slovenia; jagodic.sara@gmail.com

**Keywords:** food allergy, oral food challenge, anaphylaxis, reaction severity, child

## Abstract

**Highlights:**

**What are the main findings?**

- Atopic comorbidities (allergic rhinitis, asthma, atopic dermatitis) and even moderately positive allergy tests significantly increase the likelihood of an allergic reaction during oral food challenge (OFC) in children.
- No epidemiological, clinical, or laboratory parameters—including the magnitude of specific IgE levels and/or skin prick test results—can reliably predict the severity of allergic reactions during OFC.

**What are the implications of the main findings?**

- In children with suspected IgE-mediated food allergy, particularly those with atopic comorbidities, oral food challenge remains essential and represents the gold standard for diagnosis.
- Oral food challenges should be performed using a gradual protocol under strict medical supervision in well-equipped settings, as severe reactions including anaphylaxis cannot be reliably predicted.

**Abstract:**

**Background**: Food allergy is a leading cause of severe allergic reactions in children and often results in restrictive elimination diets. The oral food challenge (OFC) remains the diagnostic gold standard but is resource-intensive and carries a risk of adverse reactions. This study aimed to identify epidemiological, clinical, and laboratory predictors of OFC outcomes and reaction severity in children with suspected immediate-type food allergies. **Methods**: We conducted a retrospective review of 148 children who underwent hospital-based, open OFCs due to suspected immediate-type food reactions. Data on demographics, comorbidities, characteristics of the initial reaction, sensitisation profiles (specific IgE [sIgE], skin prick test [SPT]), and OFC outcomes were analysed. Reactions were graded using the Ring and Messmer scale. **Results**: OFC was positive in 44 of 148 children (29.7%). However, no clinical or laboratory parameters—including prior reaction severity and the magnitude of allergy test results—were associated with the severity of reactions during OFC. Comorbidities—specifically asthma, atopic dermatitis, and allergic rhinitis—were significantly associated with a positive OFC (*p* < 0.01), as were elevated sIgE levels and larger SPT wheal diameters (*p* < 0.01 for both). The optimal thresholds for predicting a positive OFC were 0.73 IU/mL for sIgE and 3.5 mm for SPT. **Conclusions**: Oral food challenge (OFC) remains essential for confirming food allergies in children. Given that the severity of reactions during OFCs cannot be reliably predicted and that low cut-off values of allergy tests were identified for predicting a positive OFC outcome, OFCs should be performed in a controlled and fully equipped medical setting, particularly in children with atopic comorbidities.

## 1. Introduction

Food allergies are a major health concern among children and adolescents, with a prevalence of approximately 5–10%. Beyond its direct effects on health and growth, food allergies substantially affect quality of life due to the risk and fear of anaphylaxis. Participation in everyday social activities, such as school excursions or eating in restaurants, may be restricted because of extensive and often overly strict elimination diets [1,2,3].

Food allergies may result from immunoglobulin E (IgE)-mediated immediate hypersensitivity, presenting with urticaria, angioedema, or anaphylaxis within minutes to hours after allergen exposure. Oral allergy syndrome (OAS), characterised by itching and swelling of the oral mucosa after the ingestion of raw fruits or vegetables in pollen-allergic individuals, is also mediated by cross-reactive IgE antibodies. Less commonly, allergic reactions to food in infants and toddlers are non-IgE-mediated and appear hours to days after ingestion due to cell-mediated or mixed-type immune responses [1,4].

In Europe, cow’s milk and hen’s egg are the most common allergens in infants and toddlers. With increasing age, peanut and tree nut allergies become more prevalent, whereas cow’s milk and egg allergies frequently resolve by school age. A considerable proportion of children allergic to cow’s milk, peanuts, or tree nuts experience severe reactions, including anaphylaxis. In contrast, sensitisation to wheat, soy, fruits, and vegetables—although relatively common—typically remains clinically silent or causes only mild reactions such as OAS. Overall, food allergy accounts for approximately two-thirds of all anaphylaxis cases in childhood [5,6].

Adverse reactions to foods are diverse, and not all are immune-mediated. Non-allergic reactions include intolerance, intoxication, pharmacological effects, and functional gastrointestinal diseases [7]. As a result, parent-reported food allergies often overestimate the true prevalence. The discrepancy is amplified by the frequent detection of clinically irrelevant sensitisation, particularly when assessing specific IgE (sIgE) blood levels. Up to 29% of children may have detectable sIgE due to cross-reactivity with pollens or sensitisation to cross-reactive carbohydrate determinants (CCDs) [3,8,9,10]. Sensitisation rates are even higher in children with atopic dermatitis, among whom up to two-thirds have sIgE to food allergens; however, only about half exhibit clinical reactions upon ingestion [11]. Component-resolved diagnostics (CRDs) improve the positive predictive value of sIgE testing, although its broader clinical application has, until recently, been mainly limited to peanut and tree nut allergy [12].

For these reasons, the European Academy of Allergy and Clinical Immunology recommends the oral food challenge (OFC) as the gold standard for diagnosing food allergies in children, especially when the diagnosis is uncertain or clinical history does not align with sensitisation testing [13]. Compared with prevalence estimates derived from parental reports or sensitisation identified by skin prick tests and/or sIgE, OFC-confirmed allergy rates are at least two-fold lower [14].

Although essential for diagnosis, the OFC is time-consuming, requires close medical supervision, and may cause significant stress due to the potential risk of severe reactions. The aim of our study was therefore to better define the indications and contraindications for OFC to reduce unnecessary procedures and minimise associated risks. To accomplish this, we sought to identify epidemiological, clinical, and laboratory characteristics in children with suspected food allergies that predict OFC outcomes.

## 2. Materials and Methods

### 2.1. Participants and Study Design

We conducted a retrospective study including all 148 children and adolescents (aged 6 months to 18 years) who underwent an oral food challenge (OFC) at our tertiary hospital between 30 June 2019 and 30 June 2024 and met the eligibility criteria. In patients who underwent OFCs to multiple food allergens and/or multiple OFCs to the same allergen, only the first challenge was included in the analysis.

OFCs were performed in children with a history of immediate-type allergic reactions (occurring minutes to a few hours after ingestion) such as urticaria, angioedema, and anaphylaxis. We also included children with a clear history of immediate reactions despite negative allergy test results.

We excluded children who avoided food solely due to a positive allergy test despite previously ingesting it without symptoms. Additional exclusion criteria were chronic or inducible urticaria, primary immunodeficiency, autoinflammatory diseases, and severe cardiac or pulmonary disorders, all considered relative contraindications to OFC. OFCs were not performed in children with a history of anaphylaxis combined with highly elevated specific IgE (sIgE) levels (≥15 U/mL for cow’s milk, ≥7 U/mL for hen’s egg, and ≥14 U/mL for peanuts) [15]. Comorbid allergic conditions, including asthma, allergic rhinitis, and atopic dermatitis, were not exclusion criteria and were recorded for statistical analysis.

### 2.2. Diagnostic Testing

Serum sIgE and skin prick tests (SPTs) were performed in all participants. Serum sIgE levels were measured using the Pharmacia CAP system (Uppsala, Sweden), with a cut-off of 0.35 IU/mL indicating sensitisation. For peanuts and hazelnuts, component-resolved diagnostics (CRDs) were performed, including sIgE to Ara h2 in 42 (82.4%) patients with a presumed peanut allergy and sIgE to Cor a9 and Cor a14 in 7 (77.8%) patients with a presumed hazelnut allergy.

SPTs were performed with commercial extracts (Lofarma SpA, Milan, Italy), using histamine as a positive control and diluent as a negative control. A wheal ≥3 mm larger than the negative control after 15 min was considered positive.

We recorded the number of foods sensitisations (polysensitisation), sIgE levels, and SPT wheal diameters. When multiple tests were available, only the most recent results before the OFC were included.

### 2.3. Oral Food Challenge Protocol

OFCs were performed in an open manner in a hospital setting according to AAAAI–EAACI PRACTALL guidelines [13]. Children were required to avoid the suspected food strictly for at least two weeks prior to the challenge. However, before this mandatory avoidance period, some children had been consuming hen’s egg or cow’s milk in extensively heated/baked form, whereas tree nuts and peanuts had been completely avoided. All chronic conditions (e.g., asthma, atopic dermatitis) had to be stable, and no acute illnesses such as infections, urticaria, vomiting, or abdominal pain were permitted at the time of the OFC.

Medications that could interfere with OFC results were withheld for the recommended periods: antihistamines for ≥7 days, systemic corticosteroids for ≥2 weeks, and bronchodilators for ≥8 h.

Initial doses were 1 g hen’s egg, 1 mL cow’s or soy milk, 0.25 g wheat flour, or 0.125 g peanuts (or peanut butter) or tree nuts. Doses were increased threefold every 30 min until a reaction occurred or a full age-appropriate serving was reached. Minimum final doses included 100 mL milk, half an egg, 25 g wheat flour, 16 whole peanuts, or 4–8 large tree nuts. All children were observed for at least two hours after completing the OFC [13].

### 2.4. Outcome Assessment

The primary outcome was the occurrence of an allergic reaction during the OFC, evaluated according to the Ring and Messmer grading scale [16]. Itching or erythema confined to the oral cavity or isolated gastrointestinal symptoms without involvement of the skin, respiratory, or cardiovascular systems were not considered allergic reactions, and the OFC was continued. Reactions were classified and recorded as anaphylaxis when they met criteria for at least Grade II (Table 1).

### 2.5. Statistical Analysis

Statistical analyses were performed using IBM SPSS Statistics version 26.0 (IBM Corp., Armonk, NY, USA). The Kolmogorov–Smirnov test was used to assess the distribution of continuous variables, including age at OFC, age at first reaction, time from the last reaction to OFC, SPT wheal size, and sIgE levels. As these variables were not normally distributed, the Mann–Whitney U test was used to analyse associations between continuous variables and OFC outcomes.

Associations between categorical variables (e.g., gender, comorbidities, type of food allergen, polysensitisation) and OFC outcomes were analysed using the chi-square test or Fisher’s exact test, as appropriate. The association between categorical variables and the severity of the allergic reaction during OFC (treated as an ordinal variable) was also assessed using the Mann–Whitney U test.

The relationship between continuous variables and the severity of the allergic reaction during OFC was evaluated using ordinal regression. Correlations between the severity of the initial allergic reaction and the severity of the OFC reaction (both treated as ordinal variables) were assessed using Spearman’s rank-order correlation.

Receiver operating characteristic (ROC) curve analysis was performed to determine optimal cut-off values of allergy test parameters for discriminating between negative and positive OFC outcomes. Statistical significance was set at α = 0.05, and all *p*-values were two-sided.

## 3. Results

### 3.1. Epidemiological, Clinical, and Laboratory Characteristics

A total of 148 children underwent oral food challenge (OFC), of whom 47 (31.8%) were girls. The age of participants ranged from 6 months to 17 years, with a median of 51 months (interquartile range (IQR)—59 months). OFCs were most frequently performed with peanuts (n = 51, 34.5%), followed by cow’s milk (n = 37, 25.0%), hen’s egg (n = 27, 18.2%), tree nuts (n = 20, 13.5%), and other food allergens (n = 13, 8.8%).

Epidemiological, clinical and laboratory characteristics of the study population are summarised in Table 2.

### 3.2. Results of Oral Food Challenge

The OFC was positive in 44 participants (29.7%). Among these, 17 children (38.6%) developed a generalised skin reaction (Grade I), 19 (43.2%) experienced a mild systemic reaction (Grade II), and 8 (18.2%) developed a severe systemic reaction (Grade III). No Grade IV reactions were observed.

All 8 children with severe systemic reactions and 2 children with mild systemic reactions (altogether 10 or 6.0%) required treatment with epinephrine. The associations between OFC outcomes and epidemiological, clinical, and laboratory parameters are summarised in Table 3.

Among participants with a positive OFC, the median age at the (most recent) original allergic reaction was 16 months (IQR—30 months) in those who experienced anaphylaxis during OFC (≥Grade II), compared with 18 months (IQR—52 months) in those with less severe reactions (*p* = 0.30). The median age at the time of OFC was 78 months (IQR—46 months) in children who developed anaphylaxis and 61 months (IQR—76 months) in those with less severe reactions (*p* = 0.43). The median interval between the original reaction and the OFC was 40 months (IQR—40 months) in children who experienced anaphylaxis and 12 months (IQR—12 months) in those with less severe reactions (*p* = 0.03).

The median sIgE level in patients who developed anaphylaxis during OFC was 3.66 IU/mL (IQR—19 IU/mL), compared with 3.53 IU/mL (IQR—15 IU/mL) in those with less severe reactions (*p* = 0.99). The median SPT wheal diameter was 5 mm (IQR—5 mm) in patients with anaphylaxis and 8 mm (IQR—7 mm) in those with less severe reactions (*p* = 0.28).

When allergy test results were analysed separately for each food allergen, the median sIgE levels in patients with a positive OFC were 1.93 IU/mL (IQR—2.0 IU/mL) for cow’s milk, 26.0 IU/mL (IQR—46.3 IU/mL) for hen’s egg, 0.31 IU/mL (IQR—0.7 IU/mL) for tree nuts, and 3.83 IU/mL (IQR—10.1 IU/mL) for peanuts. These values were compared with median sIgE levels in patients with a negative OFC of 0.52 IU/mL (IQR—2.0 IU/mL), 0.63 IU/mL (IQR—2.2 IU/mL), 0.40 IU/mL (IQR—1.1 IU/mL), and 0.41 IU/mL (IQR—6.0 IU/mL) for cow’s milk (*p* = 0.10), hen’s egg (*p* = 0.01), tree nuts (*p* = 1.00), and peanuts (*p* = 0.02), respectively.

Skin prick test (SPT) wheal diameters in patients with a positive OFC were 4 mm (IQR—6 mm) for cow’s milk, 7 mm (IQR—7 mm) for hen’s egg, 3 mm (IQR—4 mm) for tree nuts, and 8 mm (IQR—8 mm) for peanuts. In patients with a negative OFC, the corresponding wheal diameters were 0 mm (IQR—3 mm), 3 mm (IQR—5 mm), 0 mm (IQR—4 mm), and 3 mm (IQR—6 mm) for cow’s milk (*p* = 0.53), hen’s egg (*p* = 0.05), tree nuts (*p* = 1.00), and peanuts (*p* < 0.01), respectively.

When patients with anaphylaxis during OFC were compared with those with a positive OFC but without anaphylaxis, no significant differences in sIgE levels or SPT wheal diameters were observed for any of the individual food allergens. Specific IgE to the peanut component Ara h2 was measured in 42 patients (82.4%) with a presumed peanut allergy. Among patients with a negative peanut OFC, the median Ara h2 sIgE level was 0.10 IU/mL (IQR—0.7 IU/mL), whereas in those with a positive peanut OFC it was 2.45 U/mL (IQR—3.8 IU/mL) (*p* < 0.01). In patients who developed anaphylaxis during the peanut OFC, the median Ara h2 sIgE level was 1.68 IU/mL (IQR—4.7 IU/mL), compared with 3.20 IU/mL (IQR—2.3 IU/mL) in patients with a positive peanut OFC but without anaphylaxis (*p* = 0.41).

There was no significant association between reaction severity during OFC and sex (*p* = 0.45), comorbidities (*p* = 0.63), polysensitisation (*p* = 0.09), or the type of food allergen (*p* = 0.69). Likewise, there was no correlation between the severity of the original allergic reaction and the severity of the OFC reaction (ρ = 0.07, *p* = 0.64).

### 3.3. ROC Curve Analysis

**Figure 1:**
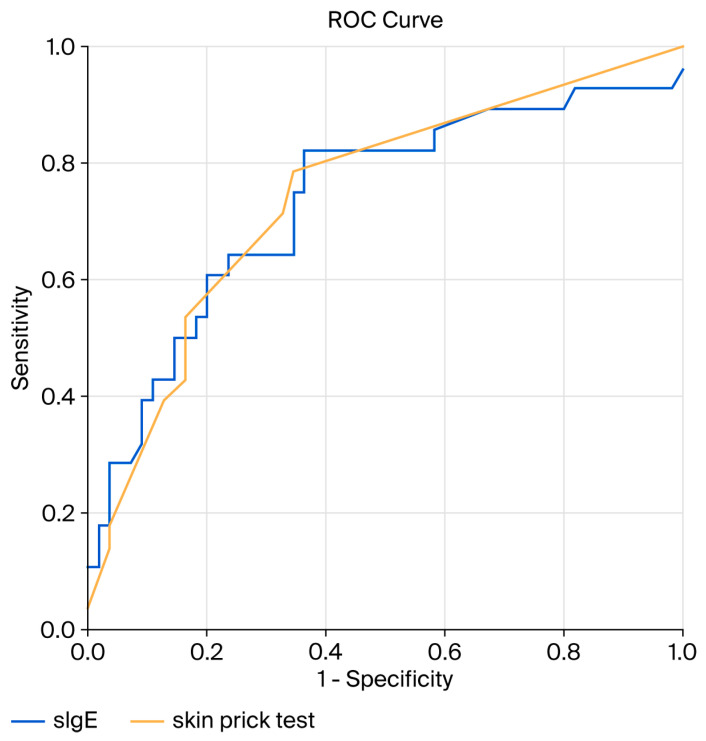
Receiver operating characteristic (ROC) curves for specific IgE (sIgE) levels and skin prick test (SPT) wheal diameter in distinguishing between positive and negative oral food challenge outcomes.

The receiver operating characteristic (ROC) curve analysis (Figure 1) identified an optimal sIgE cut-off value of 0.73 IU/mL, yielding a sensitivity of 81.8% and a specificity of 48.1% for predicting a positive OFC outcome. The area under the ROC curve (AUC) was 0.71 (95% CI: 0.60–0.79, *p* < 0.01).

For the skin prick test (Figure 1), the optimal wheal diameter cut-off was 3.5 mm (above the negative control), corresponding to a sensitivity of 78.6% and a specificity of 65.5%. The AUC for SPT was 0.74 (95% CI: 0.63–0.86, *p* < 0.01).

When ROC curve analyses were performed separately for the most common food allergens to discriminate between positive and negative OFC outcomes, a statistically significant AUC was observed only for peanut testing, namely for skin prick testing (AUC 0.80, 95% CI: 0.64–0.95, *p* < 0.01) and for sIgE to the peanut component Ara h2 (AUC 0.87, 95% CI: 0.76–0.99, *p* < 0.01). The optimal wheal diameter cut-off for peanut SPT was 3.5 mm above the negative control, yielding a sensitivity of 92.3% and a specificity of 62.5%. The optimal cut-off for Ara h2 sIgE was 0.31 IU/mL, corresponding to a sensitivity of 95.5% and a specificity of 70.0%. The AUC for whole-peanut sIgE was 0.70 (95% CI: 0.50–0.89, *p* = 0.07).

For tree nuts, the AUCs for sIgE and SPT were 0.61 (95% CI: 0.17–1.00, *p* = 0.54) and 0.53 (95% CI: 0.17–0.89, *p* = 0.88), respectively. For hen’s egg, the AUCs for sIgE and SPT were both 0.75 (95% CI: 0.50–0.99, *p* = 0.09 and 95% CI: 0.50–1.00, *p* = 0.08, respectively). For cow’s milk, the corresponding AUCs were 0.72 (95% CI: 0.32–1.00, *p* = 0.18) for sIgE and 0.71 (95% CI: 0.42–0.99, *p* = 0.21) for SPT.

## 4. Discussion

In this study, we found that children with comorbid allergic diseases—particularly asthma and atopic dermatitis—as well as those with higher sIgE levels and/or larger SPT wheal diameters had a significantly greater likelihood of a positive OFC. In contrast, and as a novel finding, none of these factors, including the magnitude of allergy test results, were associated with the severity of allergic reactions during OFC or the risk of anaphylaxis.

Interestingly, we did not confirm an association between the severity of the original allergic reaction and either the likelihood of a positive OFC or the severity of the reaction during OFC. This finding does not support the current recommendations of the American Academy of Allergy, Asthma and Immunology (AAAAI) and the European Academy of Allergy and Clinical Immunology (EAACI), which consider the severity of previous reactions to be an important element of pre-challenge risk assessment [13]. Our results also differ from those of Jacob et al., who reported an increased likelihood of a positive OFC in children with a history of anaphylaxis at original reaction [17]. Their substantially lower OFC positivity rate (12.3%) compared with ours (29.7%) likely reflects more restrictive contraindications to OFC and a smaller proportion of children with previous anaphylaxis included in their cohort. In contrast, in our study, approximately half of the participants had experienced anaphylaxis (Grade II or higher) at original reaction.

The absence of a relationship between original reaction severity and OFC outcome in our study may partly reflect the longer interval between the original reaction and the OFC, as we often postpone OFCs in children with severe reaction histories until allergy test values decrease. However, we still found that a longer interval between the original reaction and the OFC was associated with more severe reactions during the OFC. Nevertheless, the lack of association between any other clinical or laboratory variables and the severity of reactions during OFC is consistent with previous studies reporting similar observations [17,18,19].

Regarding comorbidities, we confirmed previous reports showing that asthma and atopic dermatitis are associated with positive OFC outcomes in children [15,20]. However, our study did not replicate the findings of Klim et al., who reported that, in addition to asthma and anaphylaxis during the original reaction as risk factors for OFC failure, sensitisation to multiple foods was also associated with a positive OFC result. In their study, the rate of positive OFCs was 32.2%, similar to the OFC failure rate observed in our cohort. Notably, more than 90% of their OFCs were performed with cow’s milk or hen’s egg [20]. In children with cow’s milk or hen’s egg allergy, polysensitisation often reflects a more severe and persistent atopic phenotype with a less favourable natural history, which may explain the higher proportion of positive OFCs among polysensitised children [21].

In contrast, more than half of the OFCs in our cohort were performed with peanuts or tree nuts, where polysensitisation more commonly reflects a cross-reactivity with tree pollens or sensitisation to CCD [9,22]. This may also explain why we did not observe a higher proportion of positive OFCs to nuts compared with other allergens, even though peanut and tree nut allergies less commonly resolve [23,24].

Therefore, we did not confirm the findings of Mustafa et al., who reported a higher rate of positive OFCs when performed with peanuts compared with other food allergens. They also observed a higher rate of reactions during OFC in younger children, which contrasts with our results, as the median age of children with positive OFCs in our study was significantly higher than that of children with negative OFCs [19]. This finding also probably reflects the postponement of OFCs in children considered at a higher risk of allergies. Our results partly align with those of Kennedy et al., who reported less severe reactions during OFC in infants and toddlers compared with older children [25].

We found both sIgE levels and SPT wheal diameter to be strong predictors of OFC outcomes, but not of the severity of reactions when they occurred. This aligns with the observations by Amin et al., who reported higher sIgE levels in clinically allergic children compared with sensitised but tolerant children [26]. Although the median sIgE values in their study were higher than in ours, our results are consistent with earlier findings by Perry et al., who identified an sIgE cut-off of 2 IU/mL corresponding to a 50% pass rate for OFCs to peanuts, cow’s milk, and hen’s egg [27], allergens which together accounted for more than 80% of our challenges.

Our ROC curve analysis identified an even lower optimal sIgE cut-off of 0.73 IU/mL, with a good sensitivity (81.8%) but limited specificity (48.1%). A similar predictive performance was observed for SPT, with an optimal wheal diameter cut-off of 3.5 mm. In our cohort, SPT was non-inferior to sIgE testing for predicting OFC outcomes, differing from the findings of Perry et al. who reported sIgE as slightly superior [27]. However, our results align with Mehl et al., who found SPT equally informative and recommended performing both tests before OFC [28].

When allergy tests were analysed separately for individual food allergens in our study, sIgE and SPT to hen’s egg and peanuts—particularly sIgE to the peanut component Ara h2—remained strong predictors of OFC outcomes, but not of reaction severity. In contrast, allergy tests for tree nuts and cow’s milk were not informative in this regard. Furthermore, when ROC curve analyses were performed separately for each food allergen, an even higher sensitivity (95.5%) and specificity (70.0%)—compared with pooled sIgE testing—were observed for sIgE to the peanut component Ara h2, using a cut-off value of 0.31 IU/mL to discriminate between negative and positive OFC outcomes. Therefore, determining the sIgE to the Ara h2 peanut component can replace OFC to peanuts in most cases; however, this approach cannot be applied to other food allergens.

Severe reactions (Grade III) occurred in eight children, and epinephrine was administered in 10 children (6.8%) during OFC. This rate was slightly higher than the 4% reported by Itazawa et al. in a large prospective study in which most OFCs involved wheat, cow’s milk, and hen’s egg [29]. The higher rate of anaphylaxis in our study can be partially explained by our low threshold for diagnosing it, as we classified reactions as anaphylaxis when they met the criteria for Grade II according to the Ring and Messmer scale—even in the absence of dyspnea—which would not universally be considered anaphylaxis under newer frameworks such as the World Allergy Organization (WAO) classification [16,30]. However, at the beginning of the inclusion period of our study in 2019, the WAO grading system had not yet been published; therefore, all patients—including the classification of their initial reactions and reactions occurring during OFC—were classified at first contact and prospectively according to the Ring and Messmer scale. Although our patients are now routinely assessed using both scales, we considered a retrospective classification of a subset of patients according to the WAO scale to be methodologically inappropriate. Our study has several limitations, most of which arise from its retrospective design. First, the classification of the severity of the original allergic reaction was based primarily on parental history, which may introduce recall bias. In addition, parents undoubtedly participated in the decision-making process regarding whether an OFC should be performed, which may have influenced case selection. Second, the relatively small sample size likely limited the usefulness of our analyses when performed separately for individual food allergens; therefore, the generalised findings of our study may not apply equally to all major food allergens. This is also reflected in the separate ROC curve analyses, where statistical significance was lost for individual allergens—except for peanut SPT and sIgE to the peanut component Ara h2—compared to the ROC analysis performed for all allergens combined. Regarding originality, previous studies have already demonstrated an association between atopic comorbidities and the magnitude of allergy test results, on the one hand, and a positive outcome of oral food challenge testing, on the other [17,19,20]. Our more original finding is the lack of association between epidemiological, clinical, and laboratory characteristics—including sIgE levels and skin prick test wheal size—and the severity of allergic reactions in children with a positive oral food challenge.

## 5. Conclusions

To conclude, OFC remains the gold standard for diagnosing food allergies in children, except for peanut allergies, where component resolved diagnostics—particularly sIgE to Ara h2—can replace OFC in most cases. OFCs should preferably be performed in a hospital setting, especially in children with comorbidities such as asthma or atopic dermatitis and clearly positive allergy test results. Notably, and representing the key finding of this study, neither clinical characteristics, including the original reaction severity, nor laboratory parameters, including the magnitude of allergy test results, were able to predict the severity of reaction at OFC when it occurs. This underscores the inherent unpredictability of OFC outcomes and the need for full preparedness for severe reactions in all challenged patients.

## Figures and Tables

**Table 1:** Ring and Messmer grading scale for allergic reactions [16].

Grade	Skin	Gastrointestinal Tract	Respiratory Tract	Cardiovascular System
I	Flushing; itching; urticaria; angioedema	No symptoms	No symptoms	No symptoms
II	As in Grade I, plus at least one of the following:	Nausea; vomiting; abdominal cramps	Hoarseness; dyspnea	Tachycardia (increase ≥ 20 beats/min); hypotension (decrease in systolic blood pressure ≥ 20 mmHg); arrhythmia
III	As in Grade I, plus at least one of the following:	-	Laryngeal edoema (stridor); bronchospasm; cyanosis	Shock
IV	As in Grade I, plus at least one of the following:	-	Respiratory arrest	Circulatory arrest

**Table 2:** Epidemiological, clinical, and laboratory characteristics of children undergoing oral food challenge.

Quantitative Characteristic	Frequency (n)	Percentage (%)
Female sex	47	31.8
Comorbidities (asthma, atopic dermatitis, allergic rhinitis)	108	73.0
Polysensitisation ^1^	112	75.7
Grade of original allergic reaction ^2^:		
- I	74	50.0
- II	49	33.1
- III	24	16.2
- IV	1	0.7
**Quantitative Characteristic**	**Median**	**IQR**
Age at original allergic reaction (months) ^3^	23	41
Age at oral food challenge (months)	54	62
Interval from original reaction to oral food challenge (months)	20	31
Specific IgE serum levels (IU/mL)	1.3	6
Skin prick test wheal diameter (mm) ^4^	3	7

^1^ Polysensitisation was defined as sensitisation to more than one food allergen. ^2^ Grade of the original allergic reaction was classified according to the Ring and Messmer scale [16]. If multiple reactions occurred to the same allergen, the most recent reaction was used. Children undergoing OFC solely due to positive allergy testing had no documented original reaction. ^3^ If more than one reaction occurred, the most recent was considered. ^4^ Wheal diameter corresponds to the size of the food allergen wheal minus the diameter of the negative control.

**Table 3:** Association of epidemiological, clinical, and laboratory characteristics with the result of oral food challenge in children.

Quantitative Characteristic [n (%)] ^1^	Positive OFC 44 (29.7)	Negative OFC 104 (70.3)	*p* Value, (OR; 95% CI)
Female sex (n = 47)	9 (19.2)	38 (80.8)	0.08 (0.45; 0.19–1.03)
Comorbidities (asthma, atopic dermatitis, allergic rhinitis) (n = 108)	39 (36.1)	69 (63.9)	**<0.01** (3.96; 1.43–10.93)
Polysensitisation ^2^ (n = 112)	37 (33.0)	75 (67.0)	0.14 (2.04; 0.82–5.10)
Grade of original allergic reaction ^3^:			
- I (n= 74)	21 (28.4)	53 (71.6)	0.34
- II (n = 49)	13 (26.5)	36 (73.5)	
- III (n = 24)	9 (37.5)	15 (62.5)	
- IV (n = 1)	1 (100)	0	
Allergen ^4^:			
- Peanuts (n = 51)	22 (43.1)	29 (66.9)	0.09
- Tree nuts (n = 20)	5 (20.0)	15 (80.0)	
- Cow’s milk (n = 37)	7 (18.9)	30 (81.1)	
- Hen’s egg (n = 27)	8 (29.6)	19 (70.4)	
**Quantitative Characteristic [median (IQR)]**			
Age at original allergic reaction (months) ^5^	16 (51)	20 (37)	0.92
Age at oral food challenge (months)	68.5 (52)	45.5 (60)	**0.03**
Interval from original reaction to oral food challenge (months)	30.5 (42)	17 (27)	**0.02**
Specific IgE serum levels (IU/mL)	3.6 (16)	0.8 (2)	**<0.01**
Skin prick test wheal diameter (mm) ^6^	6 (5)	0 (0)	**<0.01**

^1^ Number of participants in each category, with percentage in parentheses. ^2^ Polysensitisation was defined as a positive allergy test to more than one food allergen. ^3^ Grade of the original allergic reaction was obtained from patient or caregiver history and classified according to the Ring and Messmer grading scale [16]. When more than one reaction to the same food allergen occurred, the most recent reaction was used. ^4^ In 13 participants, OFC was performed with allergens other than peanuts, tree nuts, cow’s milk, or hen’s egg; these participants were not included in this line of analysis. ^5^ When more than one allergic reaction occurred to the same food allergen, the most recent reaction was considered. ^6^ Skin prick test wheal diameter represents the size of the wheal for the tested food allergen minus the wheal diameter of the negative control. In participants with a negative OFC, the median SPT wheal diameter was 0 mm, while the mean was 2.5 mm. IQR—interquartile range; IgE—immunoglobulin E; OR—odds ratio; CI—confidence interval.

## Data Availability

The raw data used in this study are openly available on 9 December 2025 at https://www.kaggle.com/datasets/vojkoberce/oral-food-challenge/data.

## Abbreviations

The following abbreviations are used in this manuscript:

OFC	Oral Food Challenge
SPT	Skin Prick Test
OAS	Oral Allergy Syndrome
CCDs	Cross-Reactive Carbohydrate Determinants
CRDs	Component-Resolved Diagnostics
ROC	Receiver Operating Characteristic
IQR	Odds Ratio
CI	Confidence Interval
AUC	Area Under the ROC Curve
AAAAI	American Academy of Allergy, Asthma and Immunology
EAACI	European Academy of Allergy and Clinical Immunology
WAO	World Allergy Organization
